# Perceptual Space of Superimposed Dual-Frequency Vibrations in the Hands

**DOI:** 10.1371/journal.pone.0169570

**Published:** 2017-01-12

**Authors:** Inwook Hwang, Jeongil Seo, Seungmoon Choi

**Affiliations:** 1 Realistic Broadcasting Media Research Department, Electronics and Telecommunications Research Institute (ETRI), Daejeon, Republic of Korea; 2 Haptics and Virtual Reality Laboratory, Department of Computer Science and Engineering, Pohang University of Science and Technology (POSTECH), Pohang, Gyungsangbuk-do, Republic of Korea; University of Sussex, UNITED KINGDOM

## Abstract

The use of distinguishable complex vibrations that have multiple spectral components can improve the transfer of information by vibrotactile interfaces. We investigated the qualitative characteristics of dual-frequency vibrations as the simplest complex vibrations compared to single-frequency vibrations. Two psychophysical experiments were conducted to elucidate the perceptual characteristics of these vibrations by measuring the perceptual distances among single-frequency and dual-frequency vibrations. The perceptual distances of dual-frequency vibrations between their two frequency components along their relative intensity ratio were measured in Experiment I. The estimated perceptual spaces for three frequency conditions showed non-linear perceptual differences between the dual-frequency and single-frequency vibrations. A perceptual space was estimated from the measured perceptual distances among ten dual-frequency compositions and five single-frequency vibrations in Experiment II. The effect of the component frequency and the frequency ratio was revealed in the perceptual space. In a percept of dual-frequency vibration, the lower frequency component showed a dominant effect. Additionally, the perceptual difference among single-frequency and dual-frequency vibrations were increased with a low relative difference between two frequencies of a dual-frequency vibration. These results are expected to provide a fundamental understanding about the perception of complex vibrations to enrich the transfer of information using vibrotactile stimuli.

## Introduction

Vibrotactile stimuli are widely used to deliver various forms of information in mobile devices, such as an event alarm, feedback for a user’s input, and special effects. Mobile device vendors have mostly utilized simple sinusoidal vibrations, which can be differentiated by their frequency and amplitude. Current mobile devices can produce a very limited number of vibrotactile signals that can be distinguished by users to deliver simple information. This is because the equipped miniature vibration actuators have a very limited performance in terms of frequency bandwidth and output magnitude. Therefore, the sensations of mobile device vibrations are limited to a smooth feeling due to their high frequencies (>100 Hz) and low amplitudes [1].

In today’s era, users receive extensive information from their mobile devices. The quantity of information that is delivered to a user is also greatly increasing due to the development of new applications and the improvement of mobile device performance. Using complex vibrations can enhance the information that is transferred to develop richer expressions for vibrotactile rendering. A complex vibration can be created by a superposition of two or more sinusoidal vibration components with different frequencies. These complex vibrations can be differentiated by the frequencies and amplitudes of their components.

Dual-frequency vibration is one of the simplest forms of complex vibrations. Several recent studies demonstrated that superimposed dual-frequency vibrations have different perceptual characteristics than those of single-frequency vibrations [2–6]. Hence, use of superimposed vibrations is expected to increase the perceived diversity of vibration stimuli in a mobile device. Recently, we utilized a rough sensation of dual-frequency vibrations to express the bass beats in music [1, 7] and emphasize intensive sound effects in movies and games [6]. However, fundamental research that defines the perceptual characteristics of superimposed vibrations has not been performed.

In this study, we aimed to expand our knowledge of the perceptual characteristics of complex vibrations, which may allow for more efficient information transfer in mobile devices. To this end, we investigated the perceptual space of dual-frequency vibrations and single-frequency vibrations by measuring the dissimilarities between various vibration stimuli. The effects of various factors in complex vibrations were analyzed in this perceptual space. The perceived intensities of the dual-frequency vibrations were also measured and compared with those of the single-frequency vibrations. We report several perceptual characteristics of dual-frequency vibrations that may be utilized to design expressive vibrotactile stimuli for mobile devices.

### Vibrotactile Perceptual Space

The perceptual space of sensory stimuli can be estimated by psychophysical measurements of dissimilarities among the stimuli. Multi-dimensional scaling (MDS) is a widely used method to derive a Euclidean perceptual space from a matrix of the dissimilarities [8]. In the perceptual space, each stimulus condition is represented as a point that is located relative to the other stimulus conditions. A distance between two points corresponds to the measured perceptual distance between a stimulus pair in the dissimilarity matrix. The point coordinates are relative to each other and free from rotational or translational transforms. Using the perceptual space, perceptual criteria that classify different stimuli can be observed visually, and the relationship to physical factors of the stimuli can be analyzed.

Several attempts have been made to reveal the perceptual space of vibrotactile stimuli. Hollins et al. investigated the perceptual space of tactile stimuli using 17 real textures [9]. Three adjective pairs were plotted for the 17 textures (rough-smooth, soft-hard, and sticky-slippery) as three axes in 2D and 3D perceptual spaces. One of our previous studies estimated a perceptual space of single-frequency vibrations [10]. The vibration stimuli were presented in the hands of participants via a mobile device mockup that was vibrated with a desktop shaker in a mechanically grounded design. In the perceptual space, two dimensions that spanned a low frequency range (40–100 Hz) and a high frequency range (100–250 Hz) were almost orthogonal. A bipolar adjective rating for 13 adjective pairs was also conducted, and the results were projected on the perceptual space to show the change of qualitative feelings of single-frequency vibrations. Ternes et al. conducted an MDS analysis of 84 vibrotactile icons [11]. In this study, a cluster sorting method was used, rather than the conventional pairwise comparison method, to derive the dissimilarity matrix for MDS [12]. The icons were made of various rhythms that were presented using a piezo-mounted handheld touch screen. Two axes of ‘even-uneven’ and ‘short notes-long notes’ were found as principle dimensions in the perceptual space of these tactile icons. Recently, Okamoto et al. found five dimensions of tactile perception through the meta-analysis of previous studies from other researchers [13]. The dimensions were macro roughness, fine roughness, friction, warmness, and hardness. They analyzed previous studies about real textures rather than synthesized vibratory stimuli. For vibration stimuli, only macro and fine roughness can be accounted for as their characteristic factors.

The previous studies presented some clues for understanding the percepts of vibrations using perceptual spaces. However, more studies are needed to unveil the fundamental characteristics of vibratory perception.

### Superimposed Vibration

A superimposed signal can be made by adding two or more sinusoidal signals with different frequencies. Recent studies have investigated the perceptual characteristics of the superimposed vibrations, which are different from simple sinusoidal vibrations [2–4]. Bensmaia et al. proposed a perceived intensity model for Pacinian mediated vibrations [2]. The model assumed the perceived intensity of a superimposed vibration as an arithmetic sum of the perceived intensity of each component. They estimated a dissimilarity between the perceived intensities of the two vibrations, based on the critical band filter theory [14]. The estimated dissimilarity showed correlation coefficients (*R*^2^) of 0.77–0.79 with the measured discriminability of stimulus set. A previous study from our group reported the perceptual space of amplitude-modulated vibrations [4]. Amplitude-modulated signals are a special form of superimposed signals. Seven modulation frequencies within the frequency range of 1–80 Hz were tested with a 150 Hz carrier frequency. These data demonstrated that vibrations with a low modulation frequency (<10 Hz) were perceived to have the greatest difference from the sinusoidal vibrations. The perceptual effect of the spectral differences between superimposed tactile signals was also studied by Lim et al. [3]. They measured the detection threshold of tactile beat perception on a finger using five carrier frequencies and four beat frequencies. The beat detection threshold decreased with carrier frequency and did not change with beat frequency.

In these previous studies, the stimuli intensities were controlled in their physical scales, such as displacement or acceleration. Since a perceived intensity of a vibration varies with frequency, the tested stimuli might have different perceived intensities. The differences might account for the perceived dissimilarities evaluated in the previous studies. Therefore, the intensities should be controlled in their perception level for the investigation of the qualitative perceptual differences of vibrations.

Four types of afferents account for the perception of vibrotactile stimulus [15]. Muniak et al. found a relationship between the activation of a neural channel and the stimulus intensity [16]. They showed that the stimulus intensity of complex vibrations can be detected by the firing rate of afferents near the site of stimulation. They found that the relationships were different depending on the afferent type. We considered the perceived intensity of a dual-frequency vibration as 80% of the perceived intensity sum of the individual frequency components via a simple pilot test [1]. Our other study investigated the perceived roughness and intensity of dual-frequency vibrations [6]. The perceived roughness tended to increase as the mixing ratio became closer to 1:1 in acceleration amplitude for mixtures of 175 Hz and 210 Hz components. Meanwhile, the perceived intensity was minimized at the equal mixing ratio, which is not consistent with Bensmaia’s intensity model [2].

These previous studies partially disclose the perceptual characteristics of superimposed vibration. Based on their results, we can list several candidates of structural factors that determine the percepts of superimposed vibrations, such as intensity ratio between spectral components, component frequencies, the within frequency difference between components, and the within frequency ratio between components. Our current study aims to investigate the effects of these factors.

### Paper Overview

In this study, we focus on vibrotactile stimuli that can be used in mobile devices. Two psychophysical experiments were carried out to explore the perceptual characteristics of superimposed dual-frequency vibrotactile stimuli that were transmitted to the user’s hand via a mobile device. In the experiments, stimuli conditions were determined by the physical performance of miniature actuators that can be equipped in a mobile device. The miniature actuators are optimized to generate high frequency (>100 Hz) and relatively weak vibrations (<5 G) that are dominantly perceived via Pacinian corpuscles [15].

Participants evaluated dissimilarity scores among various single-frequency and dual-frequency vibration stimuli. We equalized the sensation levels of the stimuli for each participant because we wanted to find the qualitative characteristic differences among dual-frequency vibrations. Perceptual spaces of the single-frequency and dual-frequency stimuli were estimated from their dissimilarity scores. We examined the effects of structural factors on the percepts of the dual-frequency vibrations. Some of the structural factors (component frequency, frequency difference, and frequency ratio) are correlated to each other. Hence, the tested component frequencies were selected carefully to observe the individual effects of each factor in the perceptual space.

In Experiment I, we examined the effects of intensity mixture ratio between two frequency components on perceived dissimilarity. A dissimilarity score was evaluated at every pair of seven intensity mixture levels for each of three different frequency pairs. The estimated 2D perceptual spaces showed nonlinear relationships among the stimuli as to the intensity mixture level for the three frequency pairs. In Experiment II, we observed the effects of component frequencies on the perception of dual-frequency vibrations by measuring the dissimilarities between ten dual-frequency vibrations and five single-frequency vibrations. From the estimated perceptual space of the single and dual-frequency vibrations, we could determine the effects of component frequency and frequency ratio.

## Methods

In this section, we describe the experimental methods used in Experiments I and II. In Experiment I, we investigated the effects of intensity mixture ratio on the perceptual space of dual-frequency vibrations. The pairwise dissimilarity ratings of vibration stimuli were conducted to estimate a perceptual space for three frequency compositions. We tested seven levels of intensity mixture ratios and controlled the amplitude of each spectral component in a dual-frequency vibration. Experiment II was conducted to analyze the perceptual effects of frequency composition in dual-frequency vibrations. We measured pairwise dissimilarities and estimated a perceptual space in a set of various single-frequency and dual-frequency vibrations.

### Participants

Twenty respective participants (10 males and 10 females) participated in Experiment I (19–27 years old with a mean age of 21.9) and in Experiment II (19–26 years old with a mean age of 20.9). They were everyday users of mobile devices and had no known sensorimotor impairments. They signed a standard consent form before the experiment. The experiments were not harmful to people and the participants could interrupt the experiment whenever they wished. The IRB of authors’ institute exempted an ethical review for this study (PIRB-2016-E042) and the review was not forced by the local law. All participants successfully finished the experiment and were paid 40,000 KRW (approximately 36 USD) each. Their identifying information (name and national identification number including sex and birth date) was collected by the first author only for the payment and was discarded after the payment.

### Apparatus

**Fig 1:**
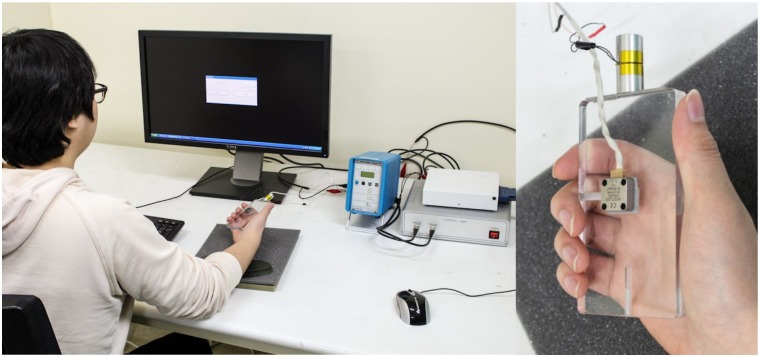
Experimental setup and posture of the participant.

Vibrotactile stimuli were transmitted to participants’ hand via a mobile device mockup that they were grasping (Fig 1). The mockup was made of acrylic resin and it was similar in size to a commercial mobile phone (11 × 6 × 1 cm). A small linear vibration actuator (Tactile Labs; Haptuator TL002-14-A; 12.5 g) generated all vibrotactile stimuli that were used in both experiments. This actuator provides stronger output over a broad bandwidth (50–500 Hz, with a weak resonance at 60 Hz) than the miniature actuators that are used in commercial mobile devices. The actuator was attached to the center of the top side of the mockup using adhesive rubber tape (Fig 1, right). A computer controlled the actuator via a 16-bit data acquisition board (National Instruments; model PCI-6251) at a 20-kHz sampling rate and a custom amplifier that supplied sufficient power for the operation of the actuator. To measure the generated vibration amplitude, an accelerometer (Kistler; model 7894A500; 7.5 g) was attached at the center of the wide face of the mockup. The acceleration data were measured at 10 kHz. The total moving mass of the mockup, the actuator, and the accelerometer was 104.5 g.

### Stimuli

Each dual-frequency vibration stimulus was made by adding two sinusoidal vibrations with different frequencies ($V_{f_{1}}\;+\;V_{f_{2}}$, *f*_1_ < *f*_2_). Their amplitudes were specified in the level of perceived intensity using our perceived intensity model that was obtained using the same mockup and vibration stimulation direction (the mockup’s height direction) [17]. The model provides an estimate of perceived intensity for a vibration stimulus from its physical amplitude and frequency. Hence, the mixture ratio of the dual-frequency vibration ($I_{f_{1}}:I_{f_{2}}$) was controlled in terms of the perceived intensity levels of the two frequency components. All stimuli were 1.5 s long to evoke stable vibrotactile sensations [18]. We used a closed-loop PD control to remove the influences of the individual-dependent hand-arm mechanical impedance and other time-varying error sources such as grip force change. The PD gain values were tuned by using the procedure described in our previous publication (see Appendix in [17]). The controller loop was executed with an update rate of 5 ms, and this rate was sufficient since we aimed to control only the amplitude of vibrations. The steady-state error of amplitude remained within 1.5%, which is significantly smaller than the difference thresholds for vibration magnitude (approximately 8% [19]).

### Experimental Conditions

In Experiment I, we used seven intensity mixture levels to generate the superimposed vibratory stimuli from two frequency components of *f*_1_ and *f*_2_: $(I_{f_{1}}:I_{f_{2}})\;=\;\left(1.0:0.0\right),\;\left(0.9:0.1\right),\;\left(0.3:0.7\right),\;\left(0.5:0.5\right),\;\left(0.3:0.7\right),\;\left(0.1:0.9\right),\;\left(0.0:1.0\right)$. At both ends of the seven conditions, (1.0:0.0) and (0.0:1.0), the vibrations are identical to the simple sinusoidal vibrations of $V_{f_{1}}$ and $V_{f_{2}}$, respectively. The experiment consisted of three sessions that differed by frequency composition $(V_{f_{1}}\;+\;V_{f_{2}}):\;V_{\text{50 Hz}}\text{ + }V_{\text{140 Hz}}\text{, }V_{\text{140 Hz}}\text{ + }V_{\text{230 Hz}}$, and *V*_50 Hz_ + *V*_230 Hz_. The three frequencies, 50 Hz, 140 Hz, and 230 Hz, were selected to represent low, medium, and high frequencies for vibrotactile stimuli in mobile devices. Similar 90 Hz steps between 50 Hz, 140 Hz, and 230 Hz were used for a comparison between each of the pairwise conditions without any harmonic relations among them.

Experiment I consisted of three sessions, a session for each frequency condition. In each session, dissimilarity was evaluated for every pair of two stimuli with different mixture ratio conditions. A session had *C*(7, 2) × 4 = 84 trials including four repetitions for each pair without considering the presentation order. A session took approximately 70–90 min including the intensity matching of stimuli. Each participant had a session each day for three days. In Experiment II, we used sinusoidal vibration components with five frequencies (50 Hz, 90 Hz, 140 Hz, 230 Hz, and 320 Hz). Ten different dual-frequency vibrations were composed by the pairwise superposition of the five frequency components. The perceived intensities of the vibration components were controlled as equal. The five frequencies were selected to consist of a Fibonacci series, *f*_*n*_ = *f*_*n*−1_ + *f*_*n*−2_, with an exception of 320 Hz (= 230 + 90 Hz), considering the frequency bandwidth of the actuator used. This frequency set design allows for better observations for the effect of within/between spectral differences in the dual-frequency vibrations and the relationship between beat frequency (*f*_2_ − *f*_1_) components and single-frequency vibrations. The pairwise dissimilarity rating was conducted for the five sinusoidal vibrations and ten dual-frequency vibrations. An experimental session for the dissimilarity rating had *C*(15, 2) × 4 = 420 trials including four repetitions for each pair without considering the presentation order. Experiment II took approximately 3.5 h, including the intensity matching of stimuli. Each participant had a session for intensity matching on the first day and a session for dissimilarity rating on the second day.

### Procedure and Data Analysis

In both experiments, participants sat in a chair and held the mockup comfortably with their dominant hand while resting their wrist on a silicon support that was placed on the table (Fig 1, left). During the trial, their response to generated vibration stimuli from the mockup was entered on a keyboard using their other hand. Participants wore earplugs to block the operating noise of the actuator.

Each experiment consisted of two steps: intensity matching and pairwise dissimilarity rating. Differences in perceived intensity between vibrotactile stimuli affect their perceived dissimilarities. Since we were concerned with the perceptual effects that were caused only by spectral differences, intensity matching was necessary to equalize the perceived intensities of the stimuli used for dissimilarity rating.

All participants were denoted anonymously by S1–S12 and their responses were collected using a GUI program. The response data were managed separately from their identifying information.

#### Intensity Matching

In the intensity matching experiment that used the method of adjustment, a participant was asked to adjust the amplitudes of vibrotactile stimuli to have the same perceived intensity as that of a reference stimulus. The reference stimulus was a 140 Hz sinusoidal vibration with a perceived intensity level of 3.5 (approximately 0.57 G acceleration) in all experiments. All vibrotactile stimuli that were to be used in the subsequent dissimilarity rating experiments were used as comparison stimuli. In each trial, the participant could adjust the amplitude of a comparison stimulus linearly in 40 perceived intensity levels by pressing the up/down keys on the keyboard. The upper limit of the amplitude was 6.0 in Experiment I. The limit was increased to 7.5 in Experiment II because of the greater variety induced from its wider frequency conditions. The step size was set to 0.15 in Experiment I and 0.1875 in Experiment II, which are less than 6% of the reference perceived intensity.

The participant was allowed to feel the reference and comparison stimuli repeatedly in free order. When the participant judged that the two stimuli had the same intensity, he/she pressed the ‘NEXT’ button on the GUI of the experimental program. The final amplitude of the comparison stimulus was stored as the PSE (Point of Subjective Equality) for that trial in a log file. Each trial took approximately 30–40 s to finish. The participants had a 20-s rest after each trial, and they were given an additional 3-min break after every 15 trials to prevent tactile adaptation.

The intensity matching experiment consisted of three sessions in Experiment I and one session in Experiment II. Each comparison stimulus randomly appeared four times without notifying the repetition to participants. The initial amplitude of a comparison stimulus was chosen randomly to be much lower or higher than the expected PSE, evenly in two sessions each. Prior to the experiment, participants were provided with procedural instructions and a 3-min training session. The measured PSEs of each comparison stimulus were averaged and used for each participant to equalize the perceived intensities of stimuli during the dissimilarity rating experiment.

#### Dissimilarity Rating

The next step was a dissimilarity rating experiment of dual-frequency superimposed stimuli. In each trial, two vibrotactile stimuli were presented sequentially with a 2-s inter-stimulus interval. After perceiving the two stimuli, the participant reported the degree of perceived difference between the two stimuli on a 0–100 scale without a reference. Participants were instructed that 0 should indicate identical stimuli and 100 should indicate very different stimuli. This pairwise comparison method allows us to achieve a high resolution for a relatively small number of stimuli in comparison to the cluster sorting method that is suitable for a larger number of stimuli [12]. The next trial started after a 2-s break. Each vibration pair was tested four times, and the presentation order of the two stimuli within a pair was balanced. Participants had a short 20-trial training session to become familiar with the experimental procedure and reporting scale. They took a rest for 3 min after every 30–35 trials.

For data analysis, the measured pairwise dissimilarities between each vibration pair were averaged over the four repetitions for each participant. Since each participant used their own dissimilarity scales within 0–100, we used the geometric mean to obtain the grand mean over the participants. We calculated an offset of each participant from the difference between the grand mean and subjective mean and scaled each participants’ rating using the offset. These standardized dissimilarity scores were used to make a dissimilarity matrix of the tested vibration stimuli. We applied parametric MDS to the dissimilarity matrix to estimate a perceptual space that represented the relative relations between the tested stimuli.

## Results and Discussion

### Experiment I

**Fig 2:**
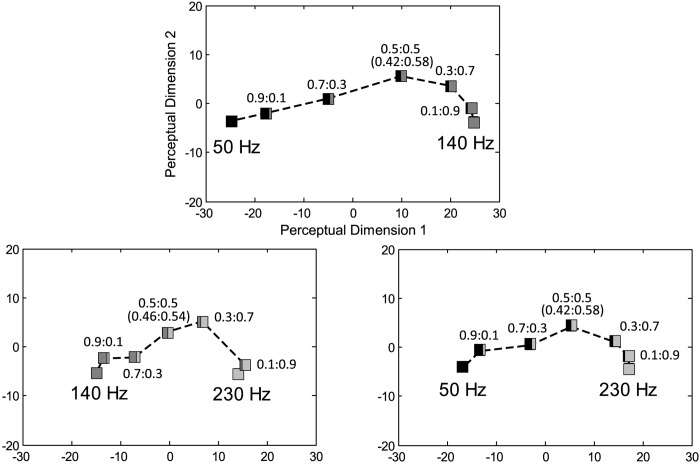
Perceptual spaces obtained from the dissimilarities measured in Experiment I. Amplitude mixture ratios in acceleration are represented in parentheses.

The averaged dissimilarity matrix for each frequency condition is represented in Table 1. We derived a two-dimensional perceptual space from each of the three dissimilarity matrices and depicted them in Fig 2. Each square box on the plot corresponds to the percept of a stimulus condition. Each box is partitioned to two regions, and their color and area represent the frequency and relative intensity of the two spectral components. A darker color represents a lower frequency and a wider area represents a larger intensity portion of the component. The distance between the two boxes corresponds to the measured dissimilarity in Table 1 with a small distortion in fitting (Kruskal’s stress formula 1 [20]< 0.04). Note that the mixture ratios are scaled in terms of perceived intensity level and acceleration amplitude (in parentheses) of each frequency component. Since the perceived intensity of vibration mostly decreases with vibration frequency [17], a higher frequency component ($V_{f_{2}}$) has a larger intensity portion when the unit is converted to acceleration amplitude.

**Table 1:** Three dissimilarity matrices of the three sets of vibration stimuli measured in Experiment. I.

*I*_50 Hz_: *I*_140 Hz_	**0.9:0.1**	**0.7:0.3**	**0.5:0.5**	**0.3:0.7**	**0.1:0.9**	**0.0:1.0**
**1.0:0.0**	5.62	18.54	37.78	48.87	49.25	49.13
**0.9:0.1**		11.79	27.45	39.41	41.62	42.19
**0.7:0.3**	11.79		14.24	23.17	29.27	30.85
**0.5:0.5**	27.45	14.24		9.67	15.35	18.37
**0.3:0.7**	39.41	23.17	9.67		7.46	7.04
**0.1:0.9**	41.62	29.27	15.35	7.46		3.28
**0.0:1.0**	42.19	30.85	18.37	7.04	3.28	
*I*_140 Hz_: *I*_230 Hz_	**0.9:0.1**	**0.7:0.3**	**0.5:0.5**	**0.3:0.7**	**0.1:0.9**	**0.0:1.0**
**1.0:0.0**	3.78	7.76	15.58	25.31	29.78	30.13
**0.9:0.1**		5.85	14.49	22.01	29.29	26.90
**0.7:0.3**	5.85		8.64	14.81	22.36	21.48
**0.5:0.5**	14.49	8.64		6.67	16.85	17.32
**0.3:0.7**	22.01	14.81	6.67		12.73	12.58
**0.1:0.9**	29.29	22.36	16.85	12.73		2.50
**0.0:1.0**	26.90	21.48	17.32	12.58	2.50	
*I*_50 Hz_: *I*_230 Hz_	**0.9:0.1**	**0.7:0.3**	**0.5:0.5**	**0.3:0.7**	**0.1:0.9**	**0.0:1.0**
**1.0:0.0**	4.94	14.34	23.63	31.88	33.59	34.04
**0.9:0.1**		9.62	19.85	27.01	30.36	31.22
**0.7:0.3**	9.62		8.74	18.13	19.84	20.46
**0.5:0.5**	19.85	8.74		8.90	13.86	14.50
**0.3:0.7**	27.01	18.13	8.90		4.38	5.56
**0.1:0.9**	30.36	19.84	13.86	4.38		2.80
**0.0:1.0**	31.22	20.46	14.50	5.56	2.80	

The numbers in the first row and column indicate the ratios of the perceived intensity levels between two frequency components.

In Fig 2, the traces of the experimental conditions are not linear and are similarly skewed close to the higher frequency component, $V_{f_{2}}$, in the three perceptual spaces. If the percepts of dual-frequency vibrations can be composed of by the linear combination of the percepts of the two frequency components, the perceptual distance between the two frequency components will be equal to the sum of the perceptual distances between the two components and their superposition. This can be represented in a perceptual space as points of stimuli aligned on a straight line between the two single-frequency vibrations. In the perceptual spaces, the percepts of dual-frequency vibrations located farther from the straight line with the increment of the weaker component. Therefore, the percepts of the seven stimuli in each of the three perceptual spaces in Fig 2 cannot be explained by the linear summations of the percepts from the two components.

We focused on this characteristic and attempted to represent the degree of perceptual difference that occurs from superposition. We calculated the maximum sum of the perceptual distances from a superimposed vibration to its two component vibrations (*d*_*sum*_ = *max*|*D*(*V*_*f*1+*f*2_, *V*_*f*1_) + *D*(*V*_*f*1+*f*2_, *V*_*f*2_)|). We also defined *d*_*base*_ as the distance between $V_{f_{1}}$ and $V_{f_{2}}$ and used it as the reference distance for each condition. The maximum sums of distances were found when the two frequency components of a dual-frequency vibration have similar intensity portions. They were 54.01 in *V*_50 Hz_ + *V*_140 Hz_, 37.17 in *V*_140 Hz_ + *V*_230 Hz_, and 38.21 in *V*_50 Hz_ + *V*_230 Hz_, while *d*_*base*_ = *D*(*V*_*f*1_, *V*_*f*2_) was larger in *V*_50 Hz_ + *V*_140 Hz_ (49.52) than those in the other two conditions (29.13 in *V*_140 Hz_ + *V*_230 Hz_, and 33.98 in *V*_50 Hz_ + *V*_230 Hz_). Since the dissimilarity scores for each condition were measured in different experimental sessions, comparison among *d*_*sum*_ should be conducted carefully. In a ratio between the two perceptual distances, *d*_*sum*_/*d*_*base*_ represents the relative maximum perceptual difference of dual-frequency superposition. We calculated and showed *d*_*sum*_/*d*_*base*_ with a frequency ratio between the two components (*f*_2_/*f*_1_) for each of the three frequency conditions in Table 2. The largest *d*_*sum*_/*d*_*base*_ (1.28) was observed in 140 Hz+230 Hz with the smallest *f*_2_/*f*_1_ (1.64). This result promises that we can achieve about 28% farther perceptual distance in total when the dual-frequency vibration is utilized in an application that requires discrimination of the stimuli. In the other two conditions, *d*_*sum*_/*d*_*base*_ was relatively small, approximately 1.1. This relationship between *d*_*sum*_/*d*_*base*_ and *f*_2_/*f*_1_ is further discussed in the results of Experiment II.

**Table 2:** Frequency ratios (*f*_2_/*f*_1_) and the maximum sums of relative maximum perceptual disparity ($\frac{d_{sum}}{d_{base}}=\frac{max|D\left(V_{f1+f2},V_{f1}\right)+D\left(V_{f1+f2},V_{f2}\right)|}{D(V_{f1},V_{f2})}$) for the three frequency conditions.

*f*_1_ + *f*_2_	**50 Hz+140 Hz**	**140 Hz+230 Hz**	**50 Hz+230 Hz**
*f*_2_/*f*_1_	2.80	1.64	4.60
*d*_*sum*_/*d*_*base*_	1.09	1.28	1.12

### Experiment II

**Fig 3:**
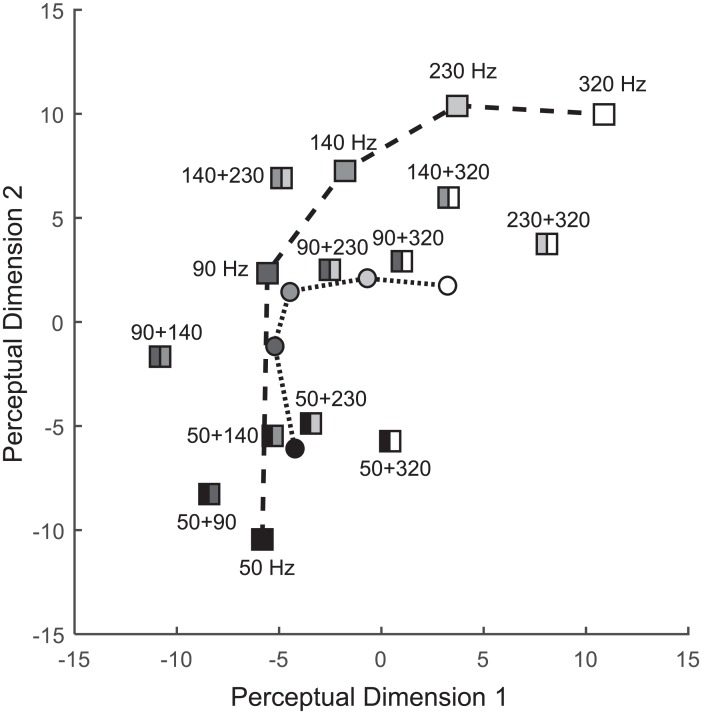
Estimated perceptual space of 15 vibration stimuli (squares) and 5 centroids for the frequency components (circles) in Experiment II.

A dissimilarity matrix of the five single-frequency vibrations and ten dual-frequency vibrations is shown in Table 3. Fig 3 shows the perceptual space estimated from the dissimilarity matrix with an acceptable distortion (Kruskal’s stress formula 1 = 0.112). In Fig 3, we also represent the centroids for vibrations containing each frequency component using circles. The single-frequency vibrations (long dashed line) and the centroids of frequency components (short dashed line) were connected respectively in the plot. They have an ‘elbow’ between 90 Hz and 140 Hz. This is similar to the 100 Hz elbow point in the perceptual space of simple sinusoidal vibrations that was observed in our previous study [10]. It is known that the Pacinian (PC) channel is the most sensitive in the perception of >40 Hz vibrations [15]. However, Rapidly Adapting (RA) channel also affects the perception of suprathreshold stimuli below 100 Hz. The elbow point near 100 Hz can be regarded as a border between low-frequency (RA channel dominant) vibration and high-frequency (PC channel dominant) vibrations [10].

**Table 3:** Dissimilarity matrix of the five single-frequency vibrations and ten dual-frequency vibrations measured in Experiment II.

**frequencies**	**90**	**140**	**230**	**320**	**50+90**	**50+140**	**50+230**	**50+320**	**90+140**	**90+230**	**90+320**	**140+230**	**140+320**	**230+320**
**50**	12.25	16.89	22.02	25.54	4.09	5.16	8.22	8.88	10.67	14.08	15.75	16.19	17.88	19.94
**90**		7.10	14.05	17.30	13.01	8.36	8.95	10.26	10.96	4.41	6.14	8.77	10.01	13.97
**140**	7.10		7.62	13.43	16.88	12.38	11.65	12.87	12.95	5.53	6.27	6.92	5.89	11.71
**230**	14.05	7.62		7.85	21.24	16.97	16.55	15.86	17.50	9.98	9.01	9.82	5.67	8.01
**320**	17.30	13.43	7.85		25.63	21.79	20.80	18.62	21.46	16.84	13.31	17.30	7.93	9.09
**50+90**	13.01	16.88	21.24	25.63		4.68	8.92	9.17	6.41	12.94	16.46	16.00	18.47	19.66
**50+140**	8.36	12.38	16.97	21.79	4.68		3.91	6.16	7.66	8.27	10.77	11.06	13.61	17.09
**50+230**	8.95	11.65	16.55	20.80	8.92	3.91		5.38	9.31	8.20	10.52	10.86	13.78	14.67
**50+320**	10.26	12.87	15.86	18.62	9.17	6.16	5.38		11.31	9.03	8.90	12.58	12.11	14.82
**90+140**	10.96	12.95	17.50	21.46	6.41	7.66	9.31	11.31		10.93	13.03	10.90	14.99	16.78
**90+230**	4.41	5.53	9.98	16.84	12.94	8.27	8.20	9.03	10.93		5.38	6.36	6.43	11.61
**90+320**	6.14	6.27	9.01	13.31	16.46	10.77	10.52	8.90	13.03	5.38		7.31	5.22	10.10
**140+230**	8.77	6.92	9.82	17.30	16.00	11.06	10.86	12.58	10.90	6.36	7.31		9.12	7.47
**140+320**	10.01	5.89	5.67	7.93	18.47	13.61	13.78	12.11	14.99	6.43	5.22	9.12		7.48
**230+320**	13.97	11.71	8.01	9.09	19.66	17.09	14.67	14.82	16.78	11.61	10.10	7.47	7.48	

The numbers in the first row and column indicate the parameters of vibrations in frequency (Hz).

We analyzed the effects of structural factors of dual-frequency vibrations on the measured perceptual dissimilarities. Component frequencies (*f*_1_ and *f*_2_), frequency ratio (*f*_2_/*f*_1_), and frequency difference (*f*_2_ − *f*_1_) can be derived from the two component frequencies.

First, we can find an effect of component frequency on the estimated perceptual space. In Fig 3, the points of the single-frequency vibrations move toward the top-right part of the plot with increasing frequency. For the dual-frequency vibrations, an increase of the component frequency resulted in the coordinates that were closer to the single-frequency vibration with a higher frequency. Since the increase of the vibration frequency means an increase of pitch, this suggests that the pitch of dual-frequency vibrations increases with their component frequencies.

In this relationship, the effect of a lower frequency component seems to be more dominant than the effect of a higher frequency component. The perceived feeling of a dual-frequency vibrations was closer to that of the single-frequency vibration of *f*_1_ than the vibration of *f*_2_. For example, the perceptual distance from *V*_50 Hz_ + *V*_230 Hz_ to *V*_50 Hz_ is 8.22, which is approximately half the distance from *V*_50 Hz_ + *V*_230 Hz_ to *V*_230 Hz_, 16.55. On average, the distance from a dual-frequency vibration of $V_{f_{1}}\;+\;V_{f_{2}}$ to the single-frequency vibration of $V_{f_{1}}$ was only 49.8% of the distance to $V_{f_{2}}$. In addition, the average distance from a single-frequency vibration to the dual-frequency vibrations that include the single-frequency component increased with frequency: 5.15 at 50 Hz, 5.83 at 90 Hz, 7.01 at 140 Hz, 9.40 at 230 Hz, and 11.57 at 320 Hz. The centroids of the dual-frequency vibrations that include the single-frequency component in Fig 3 also show this effect of component frequency. The distance between a single-frequency vibration and the centroid is closer in low frequency vibrations than those of high frequency vibrations.

**Fig 4:**
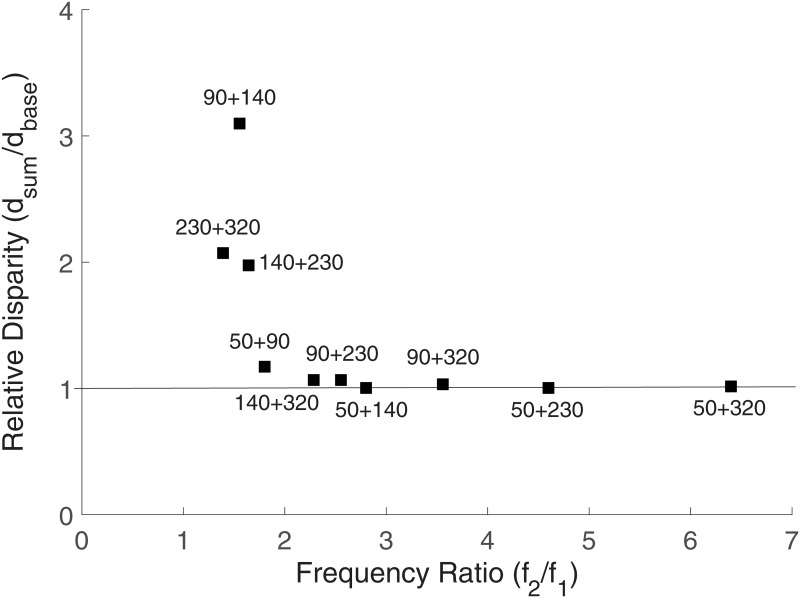
Frequency ratio (*f*_2_/*f*_1_) vs. *d*_*sum*_/*d*_*base*_ of the ten dual-frequency vibration stimuli in Experiment II.

The effect of the frequency ratio (*f*_2_/*f*_1_) can be understood by comparing the frequency ratio with the relative difference of the dual-frequency vibration from their two frequency components. In Experiment I, we calculated *d*_*sum*_/*d*_*base*_ in the perceptual spaces to observe the amount of a dual-frequency vibration that was deviated from a line connecting the two single-frequency vibrations. The relationship of *f*_2_/*f*_1_ and *d*_*sum*_/*d*_*base*_ in Experiment II is shown in Fig 4. In the plot, the values of *d*_*sum*_/*d*_*base*_ mostly decrease with *f*_2_/*f*_1_ when *f*_2_/*f*_1_ < 2.0 and are saturated when *f*_2_/*f*_1_ ≥ 2.0. When *f*_2_/*f*_1_ < 2.0, the average *d*_*sum*_/*d*_*base*_ was 2.08, which is almost twice the average of *d*_*sum*_/*d*_*base*_ (1.03) when *f*_2_/*f*_1_ ≥ 2.0. The result shows that the lower frequency ratio in a dual-frequency vibration results in a larger perceptual difference from their two frequency components. In addition, the stimuli were perceptually closer than their *f*_1_ component to the 50 Hz vibration when *f*_2_/*f*_1_ < 2.0, except the case of *V*_50 Hz_ + *V*_90 Hz_.

Our previous study found a trend of increasing consonances with the component frequencies and the frequency ratio in dual-frequency vibrations [5]. The consonance was increased with *f*_1_ and saturated when *f*_2_/*f*_1_ ≥ 2.0. The results of Experiment II are consistent with the difference of consonance scores between frequency ratio and base frequency. From the results, we can expect the value of superimposed vibrations can be utilized in expanding the perceptual space of vibration and substituting the tactile sensation of low frequency vibrations.

## General Discussion

### Perceived Intensity of dual-frequency Vibration

#### Spectral Summation

**Fig 5:**
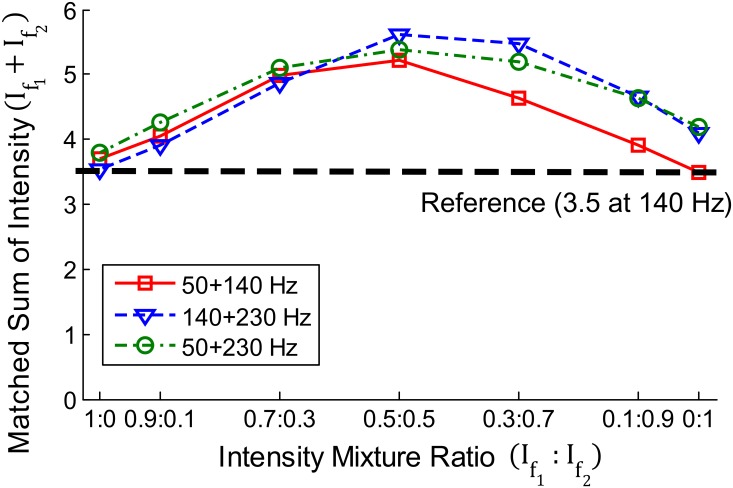
Averaged results of intensity matching in Experiment I. The arithmetic sum of the perceived intensities for the superimposed components are represented. The dotted horizontal line represents the reference perceived intensity.

In Experiments I and II, the perceived intensities of the tested vibration stimuli were equalized for each participant through the intensity matching process. Fig 5 presents the mean results obtained from the intensity matching in Experiment I. The y-axis represents the sum of the intensity levels for the frequency components of a stimulus ($I_{f_{1}}\;+\;I_{f_{2}}$). In the plot, we can observe a trend along the three frequency conditions. For the same perceived intensity, a larger sum of intensities was needed when the two frequency components were almost equal ($I_{f_{1}}:I_{f_{2}}\;=\;0.5:0.5$) in comparison to those at other ratio conditions. In the 0.5:0.5 condition, the sum of the component intensities was 5.2–5.6. In other words, the overall perceived intensity was 62.5–67.3% of the intensity sum when the two equal-intensity vibration components are superimposed. This intensity summation trend was briefly assumed and utilized in our recent studies to transfer a dual-frequency vibration at a desired intensity [1, 6]. An estimate of 62.5% was used to compute the perceived intensity of equally mixed dual-frequency vibrations from their sum of intensities in our previous study (*f*_1_ = 157 Hz, *f*_2_ = 243 Hz), which is on the lower boundary of the current results [1].

#### Error in Perceived Intensity

Error between our perceived intensity model [17] and matched intensity was observed during intensity matching. Aside from the 140 Hz vibration, which was the reference, small errors were observed at 50 Hz and errors of approximately 20% can be found at 230 Hz. The parametric fitting of the perceived intensity model is suspected to be a primary error source. The fit showed a high *R*^2^ value (>0.99) and the fitting errors near the reference intensity level (3.5) were approximately 10% at 250 Hz. This error range is smaller than the error in this study.

Another plausible error source is the difference in participants between our previous and current studies. The participants’ age in these studies were similar (mostly in their 20s). In each of these studies, the standard error among the participants was less than 10% of the perceived intensities for high frequency conditions (> 230 Hz) and we could not find an effect of outlying participants.

The difference in the experimental methods used in the two studies may also contribute to the error. Our previous study used the absolute magnitude estimation method to measure the perceived intensities of vibration stimuli. Since there was no common reference or modulus for the magnitude estimation, the results are subject to the influenced of the stimulus context effect [21]. We used the method of adjustment with a 140 Hz reference stimulus in the current study. The participants perceived a vibration repeatedly to compare the perceived intensities of the reference stimulus to a comparison stimulus. The effect of stimulus context could be eliminated via a direct comparison between the reference and comparison stimuli. However, concrete evidence for the effects of the listed error sources could not be found in this study.

### Scale of Estimated Perceptual Spaces

In Experiment I, we estimated a perceptual space for each of the three frequency conditions. Via a within-subjects design, all participants evaluated dissimilarity scores for the three sets of stimuli over three days. We compared the spanned length of each perceptual space to test the participants’ consistency in their dissimilarity rating. When we consider the dissimilarity relationship presented in Fig 3, the 230 Hz vibration should be more distanced than the 140 Hz vibration from the 50 Hz vibration. Surprisingly, the spanned length in the *V*_50 Hz_ + *V*_140 Hz_ condition was the longest (49.52) with the *V*_50 Hz_ + *V*_230 Hz_ condition the second longest (33.98) and the *V*_140 Hz_ + *V*_230 Hz_ condition was the shortest (29.13). This inconsistency in dissimilarity rating results can be attributed to the stimulus context effect in Experiment I [21]. Participants might establish their 0–100 scale for the given stimulus condition during a session. Additionally, the contrast between stimuli affected the participants’ rating criteria. Hence, the perceptual distances measured in Experiment I should not be directly compared throughout the sessions with different frequency conditions and thus should only be considered in similar frequency conditions. For the comparison among frequency conditions, a reference for dissimilarity will be needed in future studies.

## Conclusions

In this paper, we report that the perceptual spaces of dual-frequency vibrations varied in component frequency and intensity mixture ratio in a mobile mockup. This was demonstrated with two psychophysical experiments. The percepts of the dual-frequency vibrations, which can be distinguished from simple sinusoidal vibrations, were investigated thoroughly. The main findings were as follows: (1) The percepts of dual-frequency vibrations are different from those of sinusoidal vibrations and cannot be estimated in a linear interpolation of individual percepts of sinusoidal vibrations, (2) the percept of dual-frequency vibration is closer to the percept of its low frequency component than the percept of its high frequency component, and (3) the perceptual difference of dual-frequency vibrations from high frequency single-frequency vibrations mostly increases with a decrease of within frequency ratio, especially when the ratio is less than 2.0. We also demonstrated that the perceived intensity of dual-frequency vibration decreases when the intensity mixture level is close to even. The results of this study can be utilized for more expressive vibration rendering in mobile devices, such as our haptic music player [1]. We expect that further studies on perceptual characteristics on complex vibrations will improve our understanding on vibratory perception and the uses of vibrotactile feedback in mobile devices.

## Supporting Information

S1 Text Instructions and consent form used in Experiments I and II. The Korean version was used in the experiments and translated to English.
(PDF)

S1 Table Raw result of Experiment I. (XLSX)

S2 Table Raw result of Experiment II. (XLSX)
