# Supplementary material for: Perceptual Space of Superimposed Dual-Frequency Vibrations in the Hands
Source: PLoS One. 2017 Jan 12;12(1):e0169570. doi: 10.1371/journal.pone.0169570 (PMC5230860; doi:10.1371/journal.pone.0169570)
Supplement: S1 Text — The Korean version was used in the experiments and translated to English. (PDF) [file pone.0169570.s001.pdf]

실험 안내사항

1. 이 실험은 모바일 기기에서 다양한 진동의 느껴지는 차이를 알아보기 위한 실험입니다. 정해진 답은 없으므로 본인이 느끼는 대로 성실히 답해주시면 됩니다.
2. 실험은 두 파트로 나뉘어진 세션을 매일 1개씩, 3일간 수행하시게 됩니다. 세션에서의 첫 파트는 진동의 크기 조절이며, 두 번째 파트는 진동의 차이 평가입니다. 파트 사이에는 약 3분간 휴식이 있으며 소요 실험시간은 하루에 약 1시간 이내입니다.

Part 1. 같은 크기의 진동 찾기

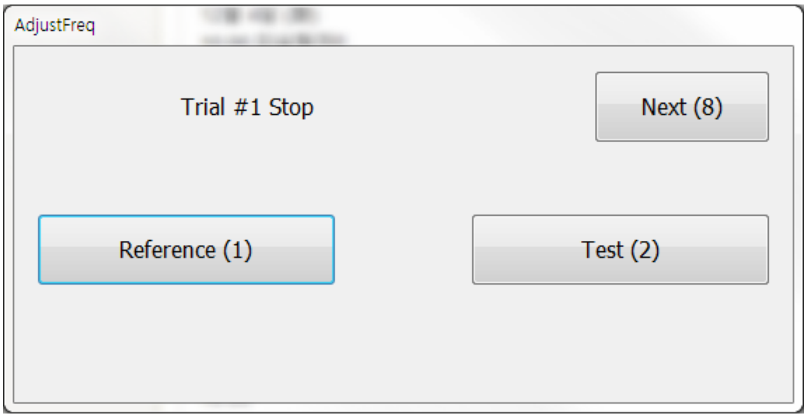

3. 첫 번째 파트의 목적은 기준 진동의 크기에 다양한 진동의 크기를 조절하여 맞추는 것입니다.
4. 프로그램에서 'Reference' 버튼을 클릭하시거나 키패드의 '1'을 누르시면 **크기가 고정된 reference 진동**이 재생되며 'Test' 버튼이나 키패드의 '2'를 누르시면 **진폭을 조절할 수 있는 test 진동**이 재생됩니다. 키보드의 상하 화살표를 누르면 test 진동의 진폭이 한 단계씩 변화합니다.
5. 두 진동이 주는 자극의 크기를 같게 조절한 후 'Next' 버튼을 누르시면 휴식메시지와 10초 이상의 휴식 뒤에 다음 조건 (trial)으로 넘어갑니다.
6. 첫번째 파트는 하루에 28개의 test 진동으로 구성됩니다.

Part 2. 진동의 차이 평가하기

7. 두 번째 파트의 목적은 순서대로 주어지는 두 진동의 차이를 숫자로 평가하는 것입니다.
8. 한 조건에서는 두 진동이 1.5초의 간격을 가지고 순서대로 주어집니다.
9. 질문이 나오면 첫 번째 진동과 두 번째 진동에서 느껴지는 차이를 0 (똑같음) – 100 (전혀 다름)의 숫자로 표현하시면 됩니다. 숫자가 클수록 큰 차이를 나타내며, 소숫점도 사용 가능합니다.
10. 진동을 잘 느끼지 못한 경우 -1을 입력하여 다시 느낄 수 있으며, 이전 조건에서 잘못 평가한 경우 -2를 입력하여 다시 평가할 수 있습니다.
11. 두 번째 파트에서는 하루에 126회의 응답을 해주시면 됩니다.
12. 매 32번마다 2분씩 쉬는 시간이 주어집니다.

공통 사항

13. 플라스틱 모델은 주로 사용하는 손으로 평소 핸드폰을 쥐는 힘과 자세로 쥐어주세요.
14. 실험에서 모든 진동의 길이는 1.5초입니다. 진동을 끝까지 느껴보고 안정된 때를 기준으로 평가해주세요.
15. 이 실험에서는 소리에 의한 영향을 막기 위해 귀마개를 착용하도록 되어 있습니다. 불편하시더라도 실험중에는 착용해주시기 바랍니다.
16. 실험 도중에 피로를 느끼시는 경우에는 중간에 잠깐 쉬는 것을 권장드립니다.
17. 실험에 참가해주셔서 감사합니다. 여러분의 실험 결과는 연구에 귀중한 자료로 사용됩니다.

동의 사항

본인은 위와 같이 실험에 대한 요구 사항들을 안내 받았으며 실험을 진행하고 연구 목적으로 필요한 정보를 제공하는 데에 동의합니다.

성명:________________________ (서명)

Introduction of Experiment

1. This experiment was designed to investigate perceived difference among various vibrations on mobile device. Since, there is no correct answer for the questions, please answer honestly as you perceive in the experiment.
2. You are asked to perform the experiment for three days, each two-parted session in a day. The first part of a session is intensity adjustment of vibration stimuli. The second part is difference assessment of vibrations. There is a three-min rest between every two parts. Expected time to finish a session is one hour.

Part 1. Intensity adjustment of vibration stimuli

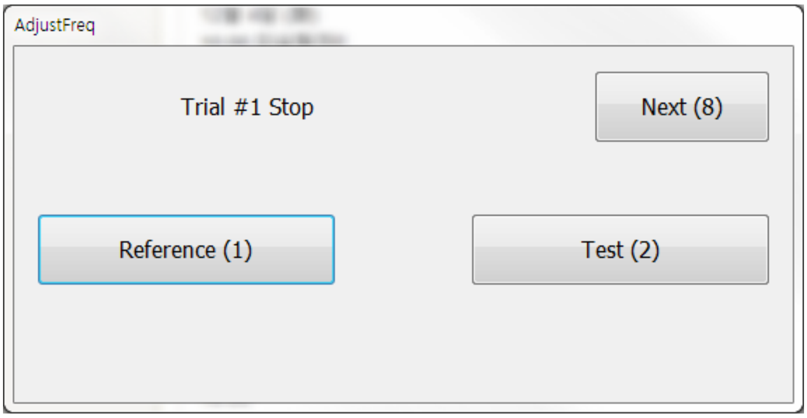

3. Purpose of the part 1 is adjust and equalize the intensity of various vibrations to the intensity of a reference vibration.
4. **A fixed reference vibration** is played when you click 'Reference' on the GUI or press '1' on the keypad. **A test vibration which can adjust its intensity** is played when you click 'Test' button or press '2' on the keypad. You can increase or decrease the intensity of the test vibration by pressing up or down arrow keys, respectively.
5. A trial can be completed after adjusting the intensity of test vibration identical to that of reference vibration. A message will be displayed when you press 'Next' button and you can move to the next trial after a 10-second rest.
6. The part 1 consisted by 28 test vibrations for a day.

Part 2. Difference assessment of vibrations

7. Purpose of the part 2 is assessing the difference of two sequentially presented vibrations in number.
8. In a trial, two vibrations are presented sequentially with 1.5 seconds of interval.
9. You are asked to evaluate the perceived difference between the first and second vibrations in a range of 0 (identical) – 100 (totally different). A larger number means a larger difference. You can use decimal points.
10. You can replay the two vibrations by typing -1 when you missed the vibrations, and you can re-evaluate the previous trial by typing -2 when you made a mistake in the previous evaluation.
11. The part 2 will be finished after 126 answers for a day.
12. A two-minute rest will be given after every 32 trials.

Common instructions

13. Please grasp the mockup in your dominant hand as like you are grasping a cell phone.
14. In this experiment, all vibrations have 1.5 seconds duration. Please feel the vibration to the end and make your answer for the most stable state.
15. You need to wear earplugs to block-out any external effect by noise. Please keep on wearing them during the experiment.
16. We recommend to take a rest in any time when you feel tired during the experiment.
17. Thank you for participating our experiment. Your experimental results are used as precious data in our research.

Consent Form

I hereby acknowledge that I have been given an opportunity to ask questions about the nature of the experiment and my participation in it. I give my consent to have data collected on my behavior and opinions in relation to the experiment for academic research. I understand I may withdraw my permission at any time.

Name:____________________ (Signature)

실험 안내사항

1. 이 실험은 모바일 기기에서 다양한 진동의 느껴지는 차이를 알아보기 위한 실험입니다. 정해진 답은 없으므로 본인이 느끼는 대로 성실히 답해주시면 됩니다.
2. 실험은 두 파트로 나뉘어져 있으며 하루에 한 파트씩 수행하시게 됩니다. 세션에서의 첫 파트는 진동의 크기 조절이며, 두 번째 파트는 진동의 차이 평가입니다. 소요 실험시간은 part 1이 1.5시간, part 2가 약 2시간 입니다.

Part 1. 같은 크기의 진동 찾기

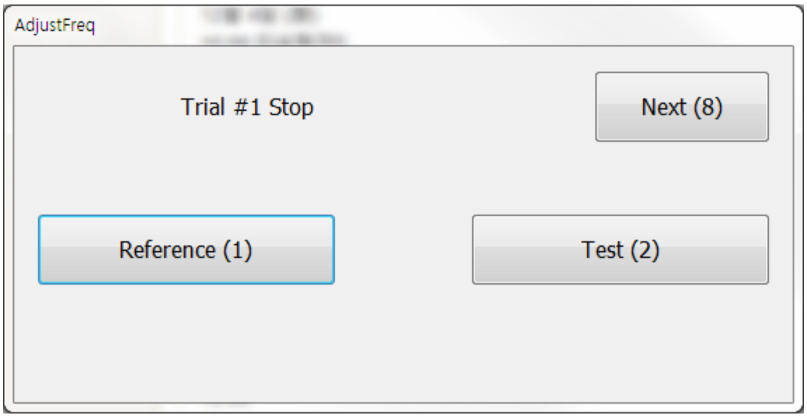

3. 첫 번째 파트의 목적은 기준 진동의 크기에 다양한 진동의 크기를 조절하여 맞추는 것입니다.
4. 프로그램에서 'Reference' 버튼을 클릭하시거나 키패드의 '1'을 누르시면 **크기가 고정된 reference 진동**이 재생되며 'Test' 버튼이나 키패드의 '2'를 누르시면 **진폭을 조절할 수 있는 test 진동**이 재생됩니다. 두 진동은 각각 자유롭게 반복하여 느낄 수 있습니다.
5. 키보드의 상하 화살표를 누르면 test 진동의 진폭이 한 단계씩 변화합니다. 총 40단계 안에서 진폭을 조절할 수 있습니다.
6. 두 진동이 주는 자극의 크기를 같게 조절한 후 'Next' 버튼을 누르시면 휴식메시지와 20초의 휴식 뒤에 다음 조건 (trial)으로 넘어갑니다.
7. 첫번째 파트는 60개의 test 진동으로 구성됩니다.

Part 2. 진동의 차이 평가하기

8. 두 번째 파트의 목적은 순서대로 주어지는 두 진동의 **크기를 제외한** 느낌의 차이를 숫자로 평가하는 것입니다.
9. 한 조건에서는 두 진동이 1.5초의 간격을 가지고 순서대로 주어집니다.
10. 질문이 나오면 첫 번째 진동과 두 번째 진동에서 느껴지는 차이를 0 (똑같음) – 100 (전혀 다름)의 숫자로 표현하시면 됩니다. 숫자가 클수록 큰 차이를 나타내며, 소숫점도 사용 가능합니다.
11. 진동을 잘 느끼지 못한 경우 -1을 입력하여 다시 느낄 수 있으며, 이전 조건에서 잘못 평가한 경우 -2를 입력하여 다시 평가할 수 있습니다.
12. 두 번째 파트는 4개의 세션으로 구성되어 있으며 각 세션마다 105회의 응답을 해주시면 됩니다.
13. 매 35번마다 3분씩 쉬는 시간이 주어집니다.

공통 사항

14. 플라스틱 모델은 주로 사용하는 손으로 평소 핸드폰을 쥐는 힘과 자세로 쥐어주세요.
15. 실험에서 모든 진동의 길이는 1.5초입니다. 진동을 끝까지 느껴보고 안정된 때를 기준으로 평가해주세요.
16. 이 실험에서는 소리에 의한 영향을 막기 위해 귀마개를 착용하도록 되어 있습니다. 불편하시더라도 실험중에는 착용해주시기 바랍니다.
17. 실험 도중에 피로를 느끼시는 경우에는 중간에 잠깐 쉬는 것을 권장드립니다.
18. 실험에 참가해주셔서 감사합니다. 여러분의 실험 결과는 연구에 귀중한 자료로 사용됩니다.

동의 사항

본인은 위와 같이 실험에 대한 요구 사항들을 안내 받았으며 실험을 진행하고 연구 목적으로 필요한 정보를 제공하는 데에 동의합니다.

성명:________________________ (서명)

Introduction of Experiment

1. This experiment was designed to investigate perceived difference among various vibrations on mobile device. Since, there is no correct answer for the questions, please answer honestly as you perceive in the experiment.
2. You are asked to perform the experiment for two days, each part in a day. The first part of a session is intensity adjustment of vibration stimuli. The second part is difference assessment of vibrations. Expected time to finish the first and second part is about 1.5 and 2 hours respectively.

Part 1. Intensity adjustment of vibration stimuli

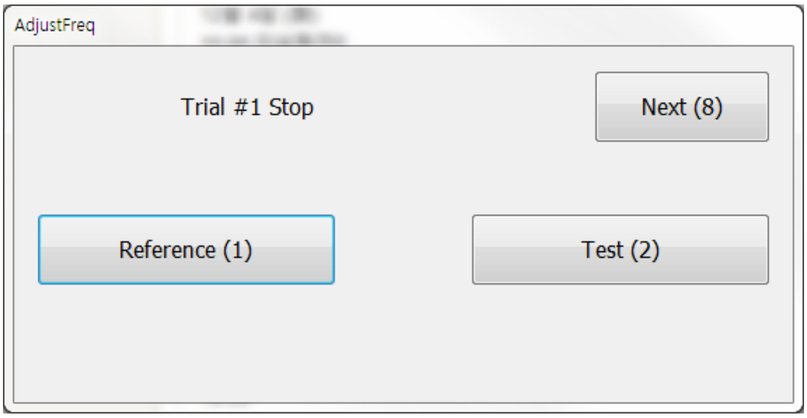

3. Purpose of the part 1 is adjust and equalize the intensity of various vibrations to the intensity of a reference vibration.
4. **A fixed reference vibration** is played when you click 'Reference' on the GUI or press '1' on the keypad. **A test vibration which can adjust its intensity** is played when you click 'Test' button or press '2' on the keypad. You can freely repeat the reference and test vibrations.
5. You can increase or decrease the intensity of the test vibration in 40 levels by pressing up or down arrow keys, respectively.
6. A trial can be completed after adjusting the intensity of test vibration identical to that of reference vibration. A message will be displayed when you press 'Next' button and you can move to the next trial after a 20-second rest.
7. The part 1 consisted by 60 test vibrations.

Part 2. Difference assessment of vibrations

8. Purpose of the part 2 is assessing the qualitative difference of two sequentially presented vibrations in number.
9. In a trial, two vibrations are presented sequentially with 1.5 seconds of interval.
10. You are asked to evaluate the perceived difference between the first and second vibrations in a range of 0 (identical) – 100 (totally different). A larger number means a larger difference. You can use decimal points.
11. You can replay the two vibrations by typing -1 when you missed the vibrations, and you can re-evaluate the previous trial by typing -2 when you made a mistake in the previous evaluation.
12. The part has 4 sessions and each session will be finished after 105 answers. .
13. A three-minute rest will be given after every 35 trials.

Common instructions

14. Please grasp the mockup in your dominant hand as like you are grasping a cell phone.
15. In this experiment, all vibrations have 1.5 seconds duration. Please feel the vibration to the end and make your answer for the most stable state.
16. You need to wear earplugs to block-out any external effect by noise. Please keep on wearing them during the experiment.
17. We recommend to take a rest in any time when you feel tired during the experiment.
18. Thank you for participating our experiment. Your experimental results are used as precious data in our research.

Consent Form

I hereby acknowledge that I have been given an opportunity to ask questions about the nature of the experiment and my participation in it. I give my consent to have data collected on my behavior and opinions in relation to the experiment for academic research. I understand I may withdraw my permission at any time.

Name:______________________ (Signature)
